# DAMPening COVID-19 Severity by Attenuating Danger Signals

**DOI:** 10.3389/fimmu.2021.720192

**Published:** 2021-08-12

**Authors:** Luis A. Silva-Lagos, Janesh Pillay, Matijs van Meurs, Alexandra Smink, Peter H. J. van der Voort, Paul de Vos

**Affiliations:** ^1^Department of Pathology and Medical Biology, University of Groningen, University Medical Center Groningen (UMCG), Groningen, Netherlands; ^2^Department of Intensive Care, University Medical Center Groningen (UMCG), University of Groningen, Groningen, Netherlands

**Keywords:** COVID-19, SARS-CoV2, TLRs, DAMPs, adenosine, cytokine storm

## Abstract

COVID-19 might lead to multi-organ failure and, in some cases, to death. The COVID-19 severity is associated with a “cytokine storm.” Danger-associated molecular patterns (DAMPs) are proinflammatory molecules that can activate pattern recognition receptors, such as toll-like receptors (TLRs). DAMPs and TLRs have not received much attention in COVID-19 but can explain some of the gender-, weight- and age-dependent effects. In females and males, TLRs are differentially expressed, likely contributing to higher COVID-19 severity in males. DAMPs and cytokines associated with COVID-19 mortality are elevated in obese and elderly individuals, which might explain the higher risk for severer COVID-19 in these groups. Adenosine signaling inhibits the TLR/NF-κB pathway and, through this, decreases inflammation and DAMPs’ effects. As vaccines will not be effective in all susceptible individuals and as new vaccine-resistant SARS-CoV-2 mutants might develop, it remains mandatory to find means to dampen COVID-19 disease severity, especially in high-risk groups. We propose that the regulation of DAMPs *via* adenosine signaling enhancement might be an effective way to lower the severity of COVID-19 and prevent multiple organ failure in the absence of severe side effects.

## Introduction

The severe acute respiratory syndrome coronavirus 2 (SARS-CoV-2) is a fast-spreading virus responsible for coronavirus disease 19 (COVID-19). In a yet-to-be-defined percentage of patients, it causes a “cytokine storm” and hypoxic respiratory failure mimicking acute respiratory distress syndrome (ARDS), which requires intensive care unit (ICU) admission and mechanical ventilation. Despite supportive care, some patients progress into multiple organ failure and death (1, 2). An important trigger for the patient deterioration might be a “cytokine storm” (1), in which high levels of interleukin 6 (IL-6) are observed (3). In COVID-19, the levels of IL-6 are associated with pulmonary complications and death (1, 2). Moreover, the blocking of the IL-6 receptor with tocilizumab might be an effective therapy for COVID-19 patients (3) and recently, the recommendations on the use of tocilizumab in critically ill COVID-19 patients have been recently updated (4).

IL-6 production and a large number of other cytokines are under the tight control of pattern recognition receptors (PRRs), among which toll-like receptors (TLRs) are the most recognized (5). The role of PRRs and TLRs in COVID-19 has received until now only minor attention. TLRs are “sentinels” that initiate inflammatory responses by binding danger- or danger-associated molecular patterns (DAMPs) (6, 7). The DAMPs can be intracellular components released by damaged virus-infected cells (7). The production of many proinflammatory cytokines mediated by TLR’s activation dependent on the nuclear factor kappa B (NF-κB) pathway. Therefore, inhibiting this pathway may limit the DAMPs/TLRs-induced cytokine storm and improve outcomes in COVID-19 patients. A “safe” alternative for this might be inhibiting NF-κB through the regulation of adenosine signaling. Adenosine is a purine nucleoside and an anti-inflammatory molecule, which signaling inhibits the TLR/NF-κB pathway (8, 9). Evidence and proof-of-principle for adenosine’s potential role in COVID-19 follow from an observational study in a small group of COVID-19 patients that received dipyridamole, an adenosine regulator, that expedited the hospital discharge and improved clinical outcomes in mild COVID-19 patients (10). In this manuscript, we reviewed the current insight on the potential role of DAMPs in COVID-19 and how these molecules might be associated with a higher COVID-19 severity in males, obese and elderly individuals. We propose and give arguments for targeting the inhibition of TLRs/DAMPs by using clinically approved adenosine signaling enhancers that may reduce the severity of COVID-19 by preventing or attenuating exacerbated inflammation, such as the “cytokine storm”.

## Coronavirus Disease 2019

### SARS-CoV-2 Infection

SARS-CoV-2, like other coronaviruses, is an enveloped, positive-sense single-stranded (ss)RNA virus with a 30 nucleocapsid of helical symmetry (11). The SARS-CoV-2 genome is 82% similar to the SARS-Cov, both causing respiratory and enteric symptoms (12). The viral entrance into human cells of SARS-CoV-2 follows from structural analysis of the virus and its receptors which suggests that the angiotensin-converting enzyme 2 receptor and transmembrane serine protease 2 can mediate viral entrance in human cells (13). Even though COVID-19 vaccination is crucial to control the current pandemic, like other diseases such as flu or influenza, COVID-19 vaccines might not reach nor protect everyone (14). Also, the threat of mutant SARS-CoV-2 that might escape from current vaccines remains a major threat. Therefore, searching for readily, available therapeutic alternatives to avoid infection, prevent viral replication, and prevent extreme immune events leading to fatal multiple organ failure is still urgently needed.

### Clinical Manifestations

The clinical manifestations of COVID-19 are diverse and complex (1, 2). Some individuals remain asymptomatic or their symptoms are self-limited. These patients represent approximately 18% of all the infected subjects (15). These patients may not directly contribute to the health system overload; however, they are capable of transmitting the virus and infect other weaker individuals (16). Once infected, clinical manifestations in symptomatic patients can vary from mild to critical disease (1). Mild disease is associated with a mild to moderate pneumonia, exhibiting symptoms similar to an upper-airway infection. Severe forms of COVID-19 are associated with dyspnea (difficulty of breathing), decreased blood hemoglobin oxygen saturation (≤ 93%), and bilateral lung opacities on chest X-ray (1). Critical forms of the disease lead to acute respiratory distress syndrome (ARDS), in which mechanic ventilation and ICU support are required. Finally, these patients can develop varying degrees of multiple organ dysfunction (1, 2). This critical phase of the COVID-19 has a high mortality, mainly in high-risk individuals, such as elderly and obese individuals (17). Noteworthy, the tight link between obesity and the susceptibility to viral infection is not specific for SARS-CoV-2 but has been reported for other viral infections, such as severe acute respiratory syndrome (SARS) caused by SARS-Cov, MERS and to a lesser degree but still significant in influenza (18, 19).

Although COVID-19 is defined as a respiratory disease, important symptoms have been reported in other systems, especially in the gastrointestinal tract (20). SARS-CoV-2 was shown to be present in feces of asymptomatic and symptomatic individuals (21) and also can enter human gut epithelial cells (22). Studies have found viral particles in feces of COVID-19 patients that were negative in nasopharyngeal swab and were discharged from the hospitals (21). The contaminated feces may be a possible way of spreading the virus but also an important route for reinfection (20). Consequently, pharmacological or dietary interventions that help to reduce the viral load or to boost the gastrointestinal immunity to avoid spreading and reinfection should also be considered.

### Risk Factors and Laboratory Findings

**Figure 1:**
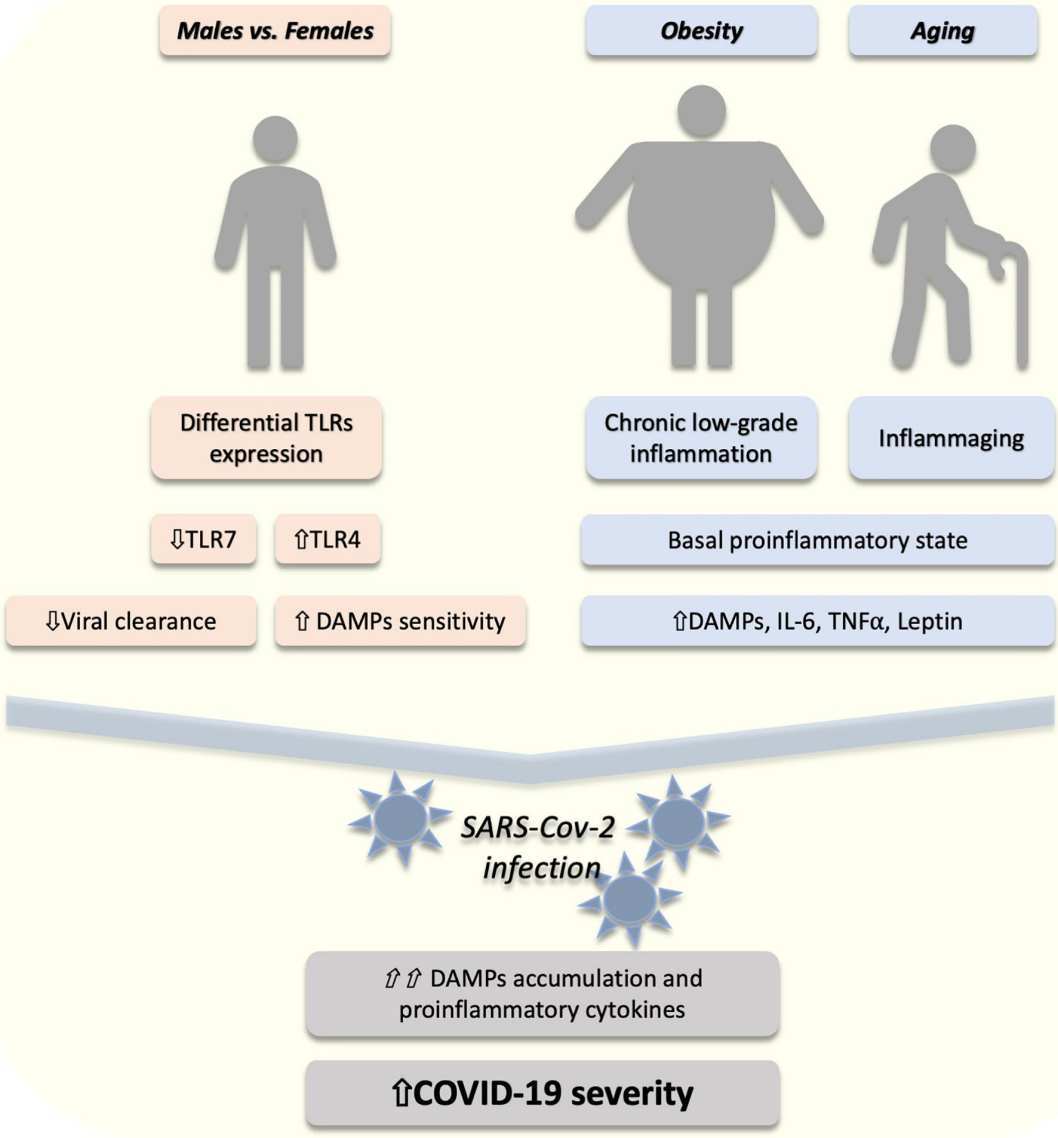
The proposed role of DAMPs/TLRs in the risk factors of COVID-19 severity. In males (compared to females) a lower (⇓) expression of toll-like receptor (TLR) 7 might (⇓) reduce the viral clearance. On the other hand, higher (⇑) TLR4 in males might lead to higher (⇑) sensitivity to danger-associated molecular patterns (DAMPs). Furthermore, obesity and aging are conditions associated with a proinflammatory state characterized by increased (⇑) DAMPs, interleukin 6 (IL-6), and tumor necrosis factor alpha (TNFα). After SARS-CoV-2 infection, an exacerbated (⇑⇑) accumulation of DAMPs and proinflammatory cytokines might explain the higher (⇑) COVID-19 severity in these individuals.

The risk factors for COVID-19 include different groups. Males seem to have a higher risk for developing more severe forms of COVID-19 (23), likely due to the lower estrogen receptors (ERs) activation and differential TLR expression in comparison to females (24). ERs are important regulators of TLRs and immune function (25). Tamoxifen, an ERs agonist, decreases the rate of SARS-CoV infection rate in female mice whose ovaries have been removed (26). Moreover, ERs activation has shown to have anti-inflammatory effects in different types of human (27) and mice (28) macrophages reducing the lipopolysaccharide (LPS)-induced activation of TLR4. Furthermore, TLR4 expression, an important DAMPs receptor (5), is higher in males (29). On the other hand, TLR7 expression, a receptor for viral structures, is higher in females (30) and regulated by ERs (31) (**Figure 1**). As will be discussed in the next section, this might explain the different responses to the virus in males and females, which might give options for therapeutic intervention. Besides, obesity is a risk factor for the severity of the disease, worsening the patient’s prognosis (32). Obesity is characterized by an imbalance in a specific family of pro and anti-inflammatory molecules, i.e. the so-called adipokines, among which leptin and adiponectin are the most recognized (33). Leptin plays a proinflammatory role in obesity and contributes to a chronic low-grade inflammation (34) with higher circulating levels of TNFα, MCP-1 and IL-6 (35). This proinflammatory state and higher circulating leptin levels might explain the poorer clinical outcomes in obese individuals infected with SARS-CoV-2 (34) (**Figure 1**). This has been also proposed for higher susceptibility in obese individuals for other respiratory viral infections, such as influenza (19). In addition to obesity, age is an important risk factor for COVID-19 (36). This might be associated with comorbidities and “inflammaging”, a phenomenon characterized by proinflammation, DAMPs accumulation, NF-κB activation and elevated levels of IL-6 and C-reactive protein (CRP) (37, 38) (**Figure 1**). All these proinflammatory cytokines are regulated by the TLRs/NF-κB pathway, suggesting an important role of this mechanism in the proinflammation associated with COVID-19.

The research reports on COVID-19 patients show lymphopenia, neutrophilia, elevated serum transaminases (alanine and aspartate transaminases), increased lactate dehydrogenase and increased CRP. Increased hypercoagulability, characterized by elevated D-dimer (fibrin fragments) and prolonged prothrombin times, has been observed in some patients (1, 39, 40). The hypercoagulability reported in COVID-19 is associated with higher mortality (41). Dysregulations in coagulation, such as upregulation of plasminogen inhibitor 1, have been previously reported in SARS-Cov infection and might also play a role in COVID-19 (41). Therefore, anticoagulant treatments might contribute to improving the clinical outcomes in COVID-19 patients (42). Plasmatic levels of IL-1β, IL-1 receptor antagonist, IL-7, IL-8, IL-9, IL-10, basic fibroblast growth factor, granulocyte-colony stimulating factor (GCSF), interferon gamma, interferon gamma-induced protein 10 (IP10), monocyte chemoattractant protein 1 (MCP1), macrophage inflammatory protein 1 alpha and beta (MIP1A and MIP1B, respectively), platelet-derived growth factor, tumor necrosis factor alpha (TNFα) and vascular endothelial growth factor levels have been reported to be higher in comparison to healthy adults (40). Furthermore, IL-2, IL-7, IL-10, GCSF, IP10, MCP1, MIP1A, and TNFα are higher in ICU patients when compared with non-critically ill patients in general ward (40). Furthermore, IL-6 levels have been associated with COVID-19 progression into severer stages and mortality (43). Many of these cytokines are controlled by the signaling of TLRs, suggesting a key role of DAMPs and these receptors in the severity of COVID-19.

## DAMPs as a Target in Managing COVID-19

The cellular damage associated with the biological cycle of the virus and the immune response in the COVID-19 patients likely increases the levels of DAMPs in the interstitial space and systemic circulation (44). DAMPs are functional molecules that participate in different cellular processes and are normally intracellularly located in specific subcellular compartments, such as the nucleus or mitochondria (6). Once the integrity of the cells is compromised, these molecules are released to the extracellular milieu, where these molecules act as a “*something-is-wrong*” or “danger” signal for the surrounding cells. For this, DAMPs can be recognized by different PRRs, among which TLRs seem to have a major role in the DAMP-induced inflammatory responses (6).

In humans, TLRs are a family of 10 members (TLR1 to TLR10) and are expressed mainly in immune cells, fibroblasts, epithelial and endothelial cells (45). TLRs act as “sentinels” sensing and recognizing specific molecular patterns associated with microorganisms (MAMPs) or DAMPS, such as high mobility group box 1, histones, S100 proteins, nuclear and mitochondrial DNA (nDNA and mtDNA) (5). TLR activation leads to intracellular activation of NF-κB pathway and the production of proinflammatory cytokines, such as IL-6, IL-1β, and TNFα (5). Furthermore, the SARS-CoV-2 viral RNA, found in different tissues and circulating in the blood, can activate TLR7 and TLR8, which are specialized in the recognition of viral RNA (5). TLR4 is an important receptor for different DAMPs (5). In males, this receptor expression is higher than in females (29). On the contrary, females express higher levels of TLR7 (30), which is specialized in virus-recognition and triggering of appropriate cellular responses, which might explain the faster clearance of the virus in females (46). A proof of principle of this are the individuals with inherited TLR7 mutations who developed an earlier and severer COVID-19 (47), confirming the importance of this receptor in the reduction of COVID-19 severity, and likely explaining the higher severity in males. This differential TLR expression, suggests that females might be more efficient in limiting the virus infection and, on the other hand, males might be more sensitive to DAMPs after cellular damage produced by the viral infection. This might explain the higher risk for a poorer outcome and more severe COVID-19 in males (**Figure 1**). Viral lysis of host-cells causes cellular and mitochondrial DAMPs to enter the circulation. Elevated plasma mtDNA, a DAMP recognized by TLR9 (48), has been associated with higher severity of ARDS in severely ill trauma and sepsis patients (49).

Hydroxychloroquine an, at the time, authorized drug for emergency use in COVID-19 that increases the pH in lysosomes altering the viral replication (10, 11). Notably, hydroxychloroquine also inhibits endosomal TLRs, such as TLR7, 8 and 9 (48, 50, 51). Also, we have shown that hydroxychloroquine is an effective TLR8 inhibitor attenuating the TLR8-induced IL-6 release (50), a cytokine associated with COVID-19 progression and mortality. However, the role of (hydroxy)chloroquine-mediated DAMPs/TLR inhibition in COVID-19 patients has not been assessed, even though the importance of endosomal TLRs in the viral clearance which are inhibited by hydroxychloroquine. Interestingly, a slower viral clearance was reported in COVID-19 patients treated with hydroxychloroquine (52). This undesired effect of hydroxychloroquine might be due to the inhibition of endosomal TLRs, such as TLR7.

The evidence against or in favor of hydroxychloroquine as a safe treatment for COVID-19 remains inconclusive. Nevertheless, the presence of different damage and viral molecules in the circulation and the interstitial space in COVID-19 (44), suggests that extracellular TLRs and DAMPs play a crucial role in inflammation induced by the cellular and tissue damage associated with SARS-CoV-2 infection (44). Therefore, DAMPs/TLRs/NF-κB signaling modulation with clinically approved drugs but with less side-effects should be considered for the treatment of COVID-19. Based on the available data, the enhancement of adenosine signaling might be a readily, fast and sound approach for modulation of DAMPs/TLRs signaling to attenuate COVID-19 severity.

## Adenosine

### Adenosine Metabolism and Signaling

Adenosine is an endogenous purine nucleoside and a drug approved for the treatment of paroxysmal supraventricular tachycardia (53). Adenosine results from the glycosidic bond between adenine and d-ribose and it is produced, released and taken up by most, if not all cells (54). Adenosine is a local regulator of cellular function, mediated by autocrine and paracrine mechanisms under normal physiological conditions and in response to acute alterations (54). Adenosine exerts diverse cellular effects mainly mediated by four G-protein-coupled adenosine receptors (ARs) (55): A_1_ and A_3_ ARs mediate the activation of G inhibitory (G_i_) protein and A_2A_ and A_2B_ ARs activate G stimulatory protein (G_s_), respectively inhibiting or activating adenylyl cyclase (cyclic adenosine monophosphate (cAMP) synthesis). ARs have different distribution and expression in different cells and tissues (56). The uptake/removal of adenosine from the extracellular space terminates the adenosine signaling (57). Adenosine uptake is mainly mediated by human equilibrative nucleoside transporters (hENTs type 1 and type 2) (57). Also, adenosine deamination mediated by extracellular adenosine deaminase (ADA) decreases adenosine concentration limiting the adenosine signaling (57). Once adenosine is inside the cell, it can be either phosphorylated by adenosine kinase or deaminated by intracellular ADA, producing AMP or inosine, respectively (55).

Adenosines half-life is approximately 10 seconds which is advantageous to prevent and control potential side-effects. Pharmacological regulation of adenosine is generally well-tolerated. Adenosine’s (and other adenosine enhancers) side-effects are usually very mild and include dizziness, flushing, and headache (around 10-20%). Severer side effects, such as ventricular arrhythmias, are reported in less than 1% of the patients. These side-effects can be solved with adenosine receptor blockers, such as aminophylline or caffeine (58, 59). The short half-life of adenosine and other adenosine enhancers, and the availability of approved adenosine receptor blockers allows that many of the side-effects are self-limited or can be controlled by the use of other drugs. However, patients should be constantly monitored for side-effects during the treatment. Furthermore, clinical trials evaluating the safety and effectiveness of adenosine enhancers in COVID-19 patients with co-morbidities (e.g., cardiovascular conditions) that can be exacerbated by adenosine signaling should be considered.

### Adenosine and DAMPs

Adenosine signaling is a strong inhibitor of the NF-κB pathway, limiting the cytokines produced by this transcription factor (60). In different cell types including immune cells, activation of A_2A_ and A_3A_ results in downregulation of the effect of TLR2, 3, 4, 7, and 9 activations and consequently to reduced release of proinflammatory cytokines, such as IL-6, IL-1β and TNFα (8, 61–63).

**Figure 2:**
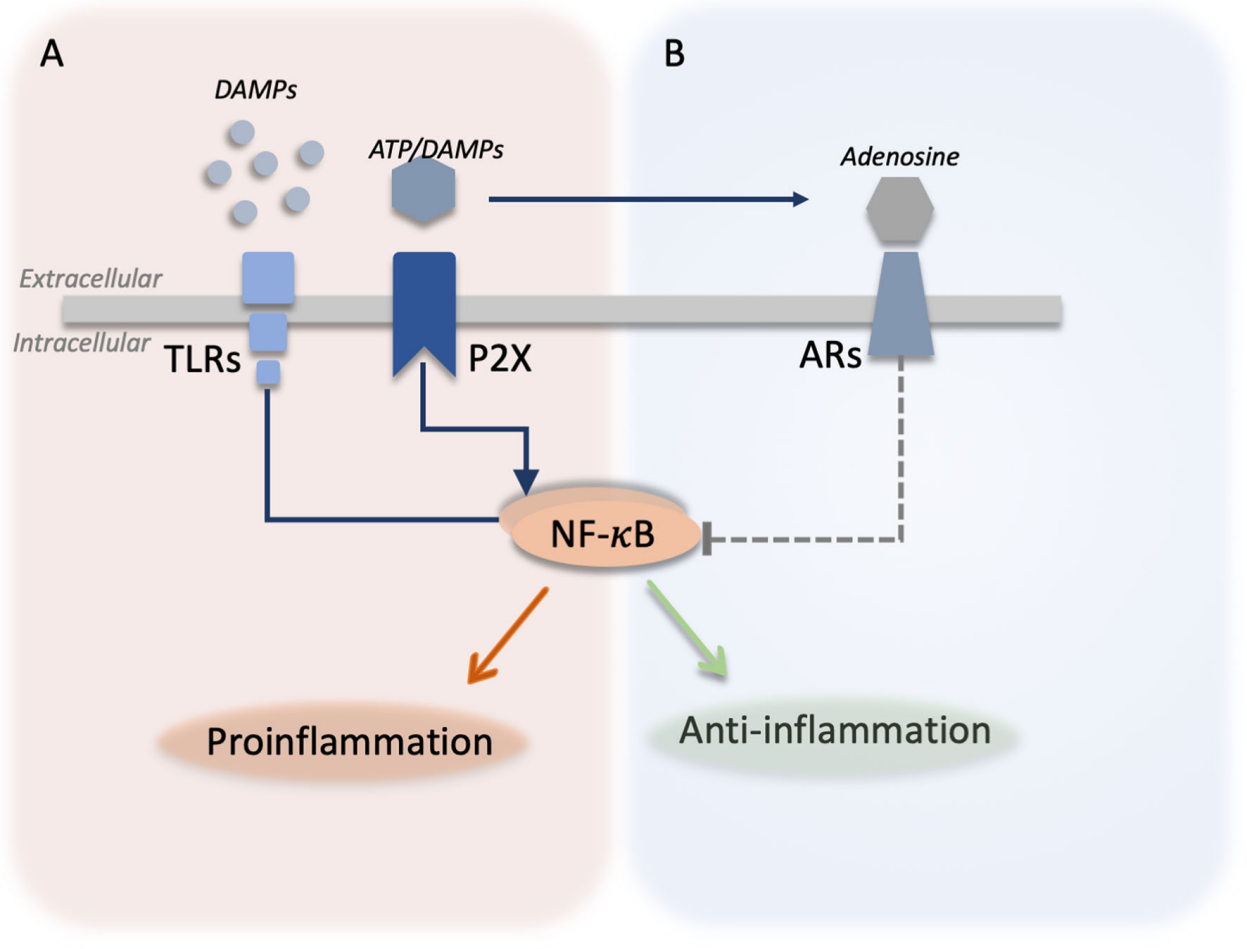
Integration between DAMPs signaling and adenosine signaling. **(A)** Different danger-associated molecular patterns (DAMPs) molecules are released by the cells and can be sensed by toll-like receptors (TLRs) leading to the activation of nuclear factor kappa B (NF-κB) and the production of proinflammatory cytokines. Similarly, adenosine triphosphate (ATP) is actively or passively released from damaged cells and activates purinergic ATP receptors (P2X) activating proinflammatory responses in the cells. ATP can be broken down by ectonucleotidases producing adenosine. **(B)** Adenosine activates adenosine receptors (ARs) and inhibits (dotted green line) TLR and P2X signaling by downregulating the NF-κB-mediated production of proinflammatory cytokines.

Adenosine can be formed from extracellular adenosine triphosphate (ATP) but as such ATP is proinflammatory molecule and a DAMP. Under stressful conditions, cells passively or actively release ATP (7). Extracellularly, ATP acts as a DAMPs signal through activation of ATP receptors (P2X, P2Y receptors) and induces activation of NF-κB (7, 64). However, extracellular ATP can be broken down into adenosine by ectonucleotidases (CD39 and CD73). Thereby, adenosine reduces the activation of NF-κB (7), counteracting the proinflammatory effects of TLR-activation and extracellular ATP signaling (65, 66). Interestingly, the activation of TLRs leads to the internalization of CD39 (66), enhancing ATP signaling and likely reducing the adenosine signaling, suggesting a tight link between adenosine and DAMPs signaling (**Figure 2**).

### Adenosine and Inflammation

Adenosine is a potent anti-inflammatory molecule that plays a crucial role in innate immunity (67). It has been proven that adenosine downregulates proinflammatory signals in different experimental models. The anti-inflammatory effect of adenosine is mediated mainly by the regulation of cAMP and the activation of cAMP-activated protein kinase (PKA) (9). As outlined above, adenosine signaling is a strong inhibitor of the NF-κB pathway (8, 9, 68). A_2A_AR signaling exerts anti-inflammatory effects on dendritic cells, neutrophils, macrophages, and T regulatory cells (69). Moreover, adenosine can reduce the activation of different TLR by inhibiting its downstream pathway, NF-κB. For this, different mechanisms have been described. A_2B_A physically binds to p105 (an NF-κB inhibitor) and decreases its degradation and by that reduces the production of proinflammatory cytokines (68). In lymphocytes from rheumatoid arthritis patients, the activation of A_2A_ and A_3_ reduced phorbol-myristate-acetate induced IL-6, IL-1β, and TNFα release (61). In murine chondrocytes, the activation of A_2A_ resulted in reduced inflammatory parameters induced by IL-1β (63). Furthermore, in systemic inflammation, the inhibition or genetic deletion of A_1_ and A_3A_ increased the systemic inflammation and mortality in a cecum ligation model of sepsis (70, 71). Similarly, A_2A_AR mediates protective effects in LPS-induced injuries in a mice model of sepsis (72). All this data strongly suggest that adenosine can decrease inflammation mediated by damage signals and TLRs activation and other proinflammatory conditions. This strongly suggests a possible beneficial effect of adenosine administration in improving the outcome in symptomatic COVID19 patients.

### Adenosine and Acute Lung Injury

Among the most recognizable threats of COVID-19 severe stages are the respiratory consequences, such as ARDS. A significant body of evidence from animal models suggests that adenosine regulation may play a crucial role in protecting lung functionality and reducing inflammation in acute lung injury models. Similarly, it seems reasonable that adenosine might be beneficial to prevent lung deterioration in COVID-19 patients, reducing the disease severity.

In the lungs, all the ARs are expressed with diverse distribution in different cell types. In human bronchial smooth muscle cells, levels of A_2B_ transcripts are highly expressed in comparison with A_1_ and A_2A_AR (73). In human lung parenchyma, A_2A_ and A_3_ expression have been reported in bronchiolar and alveolar epithelium, in bronchiolar smooth muscle cells and endothelial cells in the pulmonary arteries (74). A_1_AR expression seems to be restricted to macrophages (74) and bronchial epithelium (75). In acute lung injury models (**Table 1**), the signaling of adenosine exerts anti-inflammatory and protective effects in animal models and human cells (76). In guinea pigs (77, 86) and mice (82) with endotoxin-induced pulmonary inflammation, the administration of 2-chloroadenosine or 5’-N-ethylcarboxamidoadenosine (non-selective ARs agonists) reduced the inflammatory response and pulmonary edema. Furthermore, the administration of adenosine in pigs prevented the effects of LPS in the lungs, decreasing the extravascular lung fluid (79). Similarly, inhaled A_2A_ agonist ATL202 reduced LPS-induced neutrophil migration, microvascular permeability, and chemokine release, suggesting A_2A_AR activation as treatment of acute lung injuries complications (78). In other induced acute lung injury models, such as induced by ventilation or transplantation, the activation of A_2A_ decreased inflammation in the lungs (83–85, 87). This suggests an important therapeutic potential of adenosine signaling not only in reducing inflammation but also in maintaining the lung functionality, crucial for COVID-19.

**Table 1:** Adenosine signaling enhancement in acute lung injury animal models.

Animal	Model	Adenosine enhancers	Findings (*vs.* ALI control animals)	Reference
*Guinea pigs*	LPS induced ALI	2-chloroadenosine (non-selective ARs agonist)	⇓Tissue/plasma albumin ⇓ Lung edema ⇓ BAL macrophages ⇓ Alveolar hemorrhage ⇓ Plasma TNFα	(76)
*Mice*	LPS-induced ALI	-NECA (non-selective ARs agonist) -Adenosine	⇓ Vascular leakage ⇓ BAL protein ⇓ Lung injury score ⇓ Lung neutrophils infiltration ⇓ Weight loss ⇓ Lung IL-6 ⇓ Lung TNFα ⇓ BAL IL-6 ⇓ BAL TNFα ⇓ BAL MIP1A ⇓ BAL MIP1B ⇓ BAL IL-1β ⇓ BAL IL-2 ⇓ BAL IFNγ ⇓ BAL GCSF	(77)
*Mice*	Pulmonary ischemia-reperfusion	ATL-313 (A_2A_ receptor agonist)	⇓Pulmonary artery pressure ⇓Airway resistance ⇑ Pulmonary compliance ⇓ Lung vascular permeability ⇓ Lung edema ⇓ BAL TNFα ⇓ BAL neutrophils	(78)
*Mice*	LPS-induced lung injury	ATL202 (A_2A_ receptor agonist)	⇓ Alveolar neutrophils migration ⇓ PMN recruitment ⇓ Vascular permeability ⇓ IL-6 ⇓ TNFα	(79)
*Mice*	Ventilation-induced lung injury	-Dipyridamole (equilibrative nucleoside transporters inhibitor) -ENT2 knockout	⇑ ALI survival ⇓ Lung edema ⇑ Gas exchange ⇑ BAL adenosine ⇓ IL-6 Abolished dipyridamole effect in A_2B_ knockout	(80)
*Mice*	Pseudomonas aeruginosa-induced ALI	-NBTI (equilibrative nucleoside transporters inhibitor) -ENT1 knockout	⇑ Total lung capacity ⇑ Lung compliance ⇑ Lung elastance ⇓ Lung tissue damage ⇓ Lung edema ⇓ BAL protein level ⇓ Lung lymphocytes ⇓ Lung neutrophils ⇓Lung eosinophils ⇓ Lung TNFα ⇓ Lung IL-6 ⇓Lung IL-1β ⇑ Lung adenosine ⇓ NLRP3 activation ⇓ Caspase 20 activation	(81)
*Pigs*	LPS-induced ALI	Adenosine	Faster drop in mean arterial pressure Normalized cardiac index Delayed drop in systemic vascular resistance = Mean pulmonary artery pressure Prevents drop in right ventricular ejection fraction ⇓ extravascular lung water content = Endothelin-1	(82)
*Pigs*	Transplantation-induced ALI	ATL-146e (A_2A_ receptor agonist)	⇓ CO_2_ pressure ⇑ O_2_ pressure Prevents acidemia = Mean arterial pressure = Cardiac output index = Systemic vascular resistance = Pulmonary compliance ⇓ Pulmonary arterial pressure ⇓ Mean airway pressure ⇓Lung edema ⇓ Neutrophil infiltration ⇓ TNFα ⇓ Lung injury score	(83)
*Rats*	Nontransplantation pulmonary ischemia-reperfusion	ATL-146e (A_2A_ receptor agonist)	⇓ CO_2_ pressure ⇑ O_2_ pressure ⇓ Capillary leak ⇓ Lung neutrophils infiltration ⇓ Lung polymorphonuclear lymphocytes	(84)
*Rats*	Cardiopulmonary bypass	ATL-313 (A_2A_ receptor agonist)	= Mean arterial pressure = Cardiopulmonary bypass flow = Blood pH = CO_2_ pressure = O_2_ pressure = Blood bicarbonate ⇓ Lung IL-6 ⇓ Lung TNFα ⇓ Lung IFNγ ⇓ Lung neutrophils ⇓ Pulmonary edema (comparable to healthy animals) ⇓ Lung injury severity score (comparable to healthy animals)	(85)

⇑, Higher, increased; ⇓, Reduced, lower; =, No differences; A_2A_,A_2A_ Adenosine Receptor; A_2B,_ A_2B_ Adenosine Receptor; ALI, Acute Lung Injury; ARs, Adenosine Receptors; ATL-313, A_2A_ Adenosine Receptor agonist; BAL Bronchoalveolar lavage; CO2 Carbon Dioxide; ENT1, Equilibrative Nucleoside Transporter 1; ENT2, Equilibrative Nucleoside Transporter 2; GCSF, Granulocyte-Colony Stimulating Factor; IFNγ, Interferon Gamma; IL-1β, Interleukin 1 Beta; IL-2, Interleukin 2; IL-6, Interleukin 6; LPS, Lipopolysaccharide, MIP1A, Macrophage Inflammatory Protein 1 Alpha; MIP1B, Macrophage Inflammatory Protein 1 Beta; NBTI, S-(4-nitrobenzyl)-6-theoinosine; NECA, 5′-(N-Ethylcarboxamido)adenosine; NLRP3, NOD-, LRR- and pyrin domain-containing protein 3; O2 Oxygen; TNFα, Tumor Necrosis Factor Alpha.

Pharmacological inhibition or genetic deletion of ENTs (nucleoside transporters) in a mice model of LPS-induced lung injury resulted in increased adenosine levels, the improvement of the pulmonary barrier, and reduced lung inflammation, a phenomenon dependent on the A_2A_ and A_2B_ (80, 81). Moreover, dipyridamole (hENTs inhibitor) can bind to the SARS-CoV-2 main protease (Mpro), reducing the virus replication *in vitro* (10). This observational study also suggests that dipyridamole expedited the hospital discharge in mild COVID-19 patients, and it is associated with an improved clinical outcome (10). Another study (88) including irresponsive-to-standard therapy COVID-19 patients, showed improvement in viral clearance and reparatory capacity after use of nebulized adenosine with almost absent reported side-effects. In a similar fashion, other study (89) using nebulized adenosine inhaled adenosine (Krenosin) showed that the treated group had a significant reduction in the length of hospitalization, test positivity, CRP level and D-dimer, with significant improvement in the chest CT scans. Together, this data suggests that different strategies, for instance by using specific adenosine receptor agonists, inhibiting the adenosine uptake, or as proposed by others (90), inhibiting adenosine deamination or phosphorylation, can be used to enhance adenosine signaling, reducing the effects of acute lung injury in, for example, COVID-19 patients.

## Concluding Remarks

**Figure 3:**
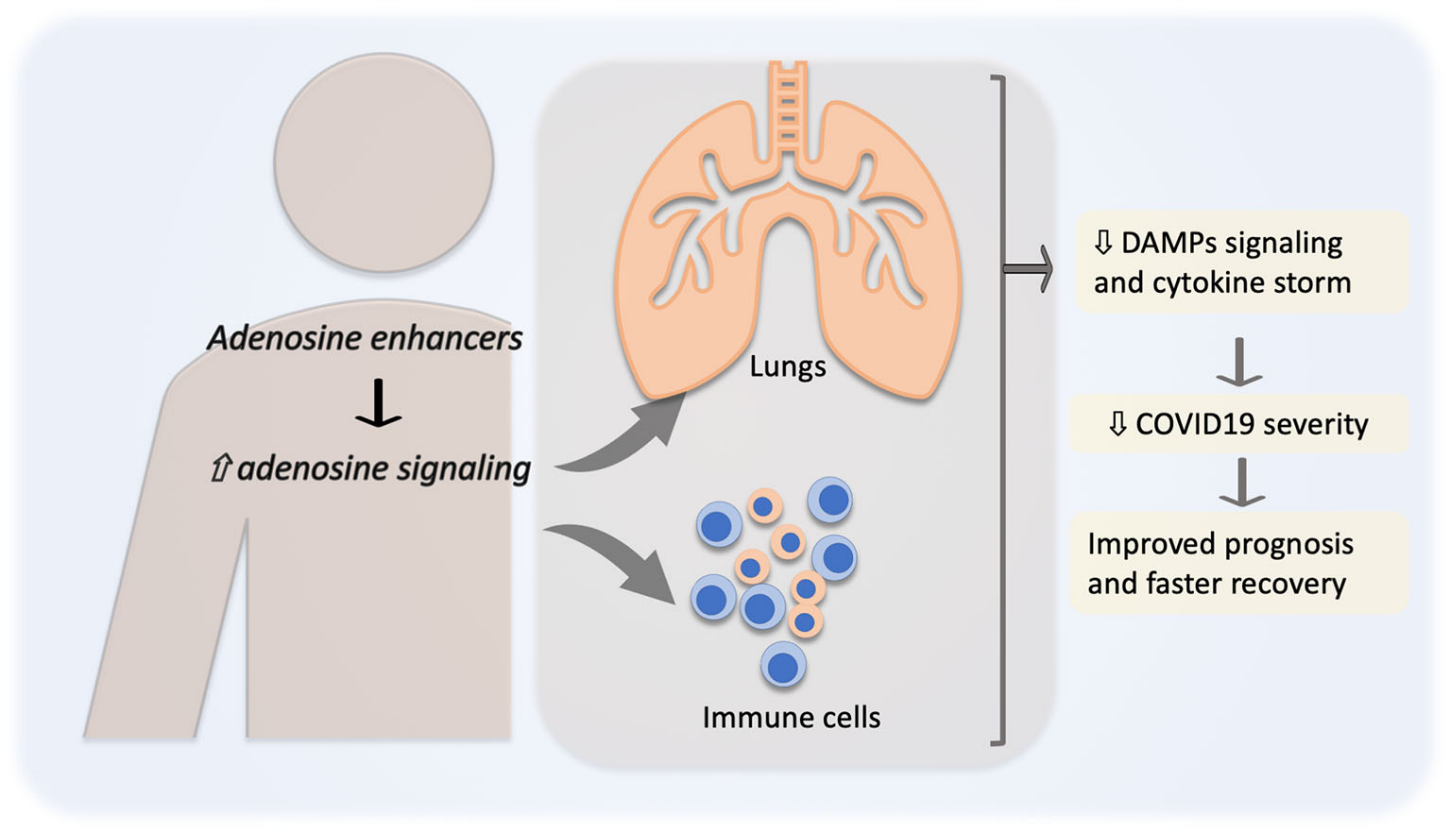
Adenosine in the reduction of COVID-19 severity. Pharmacological regulation of adenosine level and signaling, for instance by adenosine uptake inhibition or adenosine receptor agonists, will lead to adenosine signaling enhancement (⇧) resulting in a decreased (⇩) danger-associated molecular patterns (DAMPs) signaling and “cytokine storm”. All this together might result in a decreased severity and better prognosis for COVID-19 patients.

Based on the above-given line of reasoning, the regulation of DAMPs *via* adenosine signaling enhancement seems to be a promising strategy to prevent deterioration of COVID-19 patients to severe ARDS and/or multiple organ failure. Currently, there are several clinically approved drugs, such as dipyridamole or regadenoson (A2A agonist), that by different mechanisms enhance adenosine signaling. Therefore, the downregulation of DAMPs/TLRs pathway *via* the enhancement of adenosine signaling seems to be a promising, relatively safe, and clinically available therapeutic strategy for COVID-19 patients. At the moment, some of these adenosine enhancers alone or in combination (e.g., Aggrenox; Dipyridamole/Aspirin combination) are included in clinical trials to evaluate the potential benefits of adenosine regulation in COVID-19 treatment (**Table 2**). Adenosine enhancers might prevent or attenuate the SARS-CoV-2-induced “cytokine storm,” reducing the severity of COVID-19, especially in high-risk groups, lowering the health system overload and the economic burden associated with the pandemic (**Figure 3**).

**Table 2:** Clinical trials: adenosine enhancers in COVID-19 management.

Identifier	Name	Phase	Status	Completion date
*NCT04588441*	The ARCTIC Trial: Aerosolized Inhaled Adenosine Treatment in Patients with Acute Respiratory Distress Syndrome (ARDS) Caused by COVID-19	Phase 2	Not yet recruiting	Dec 2022
*NCT04424901*	Trial of Open Label Dipyridamole- In Hospitalized Patients With COVID-19 (TOLD)	Phase 2	Recruiting	May 2021
*NCT04391179*	Dipyridamole to Prevent Coronavirus Exacerbation of Respiratory Status (DICER) in COVID-19 (DICER)	Phase 2	Completed	Feb 2021
*NCT04410328*	Aggrenox To Treat Acute Covid-19 (ATTAC-19)	Phase 3	Recruiting	Dec 2021

## Author Contributions

LS-L: Conceptualization, Writing - Original Draft, Writing - Review & Editing, Visualization. JP: Data curation, Writing - Review & Editing. MM: Writing - Review & Editing. AS: Writing - Review & Editing. PHJV: Writing - Review & Editing. PV: Conceptualization, Review & Editing. All authors contributed to the article and approved the submitted version.

## Conflict of Interest

The authors declare that the research was conducted in the absence of any commercial or financial relationships that could be construed as a potential conflict of interest.

## Publisher’s Note

All claims expressed in this article are solely those of the authors and do not necessarily represent those of their affiliated organizations, or those of the publisher, the editors and the reviewers. Any product that may be evaluated in this article, or claim that may be made by its manufacturer, is not guaranteed or endorsed by the publisher.

## References

[1] Cascella M Rajnik M Cuomo A Dulebohn SC Di Napoli R. Features, Evaluation and Treatment Coronavirus (COVID-19) (2020). StatPearls Publishing. (Accessed April 7, 2020). 32150360

[2] Wu Z McGoogan JM. Characteristics of and Important Lessons From the Coronavirus Disease 2019 (COVID-19) Outbreak in China: Summary of a Report of 72314 Cases From the Chinese Center for Disease Control and Prevention. JAMA - J Am Med Assoc (2020) 323(13):1239–42. 10.1001/jama.2020.2648 32091533

[3] Zhang C Wu Z Li JW Zhao H Wang GQ. The Cytokine Release Syndrome (CRS) of Severe COVID-19 and Interleukin-6 Receptor (IL-6R) Antagonist Tocilizumab may be the Key to Reduce the Mortality. Int J Antimicrob Agents. Published Online March 29 (2020) 55:105954. 10.1016/j.ijantimicag.2020.105954 PMC7118634 32234467

[4] National Institutes of Health (NIH). Statement on Tocilizumab | COVID-19 Treatment Guidelines (2021). Available at: https://www.covid19treatmentguidelines.nih.gov/statement-on-tocilizumab/ (Accessed April 11, 2021). NIH. 34003615

[5] Kawasaki T Kawai T. Toll-Like Receptor Signaling Pathways. Front Immunol (2014) 5:461. 10.3389/fimmu.2014.00461 25309543 PMC4174766

[6] Roh JS Sohn DH. Damage-Associated Molecular Patterns in Inflammatory Diseases. Immune Netw (2018) 18(4):e27–41. 10.4110/in.2018.18.e27 PMC6117512 30181915

[7] Vénéreau E Ceriotti C Bianchi ME. Damps From Cell Death to New Life. Front Immunol (2015) 6:422. 10.3389/fimmu.2015.00422 26347745 PMC4539554

[8] Hamidzadeh K Mosser DM. Purinergic Signaling to Terminate TLR Responses in Macrophages. Front Immunol (2016) 7:74(MAR). 10.3389/fimmu.2016.00074 26973651 PMC4773587

[9] Minguet S Huber M Rosenkranz L Schamel WWA Reth M Brummer T. Adenosine and Camp are Potent Inhibitors of the NF-κb Pathway Downstream of Immunoreceptors. Eur J Immunol (2005) 35(1):31–41. 10.1002/eji.200425524 15580656

[10] Liu X Li Z Liu S Sun J Chen Z Jiang M. Potential Therapeutic Effects of Dipyridamole in the Severely Ill Patients With COVID-19. Acta Pharm Sin B (2020) 10:1205–15. 10.1016/j.apsb.2020.04.008 PMC7169892 32318327

[11] Khan S Siddique R Shereen MA Ali A Liu J Bai Q. The Emergence of a Novel Coronavirus (SARS-Cov-2), Their Biology and Therapeutic Options. J Clin Microbiol (2020) 58:e00187–20. 10.1128/JCM.00187-20 PMC7180238 32161092

[12] Yeo C Kaushal S Yeo D. Enteric Involvement of Coronaviruses: Is Faecal–Oral Transmission of SARS-CoV-2 Possible? Lancet Gastroenterol Hepatol (2020) 5(4):335–7. 10.1016/S2468-1253(20)30048-0 PMC7130008 32087098

[13] Hoffmann M Kleine-Weber H Schroeder S Schroeder S Krüger N Herrler T Erichsen S. SARS-CoV-2 Cell Entry Depends on ACE2 and TMPRSS2 and is Blocked by a Clinically Proven Protease Inhibitor. Cell (2020) 181(2):271–80.e8. 10.1016/j.cell.2020.02.052 32142651 PMC7102627

[14] Mellet J Pepper MS. A COVID-19 Vaccine: Big Strides Come With Big Challenges. Vaccines (2021) 9(1):1–14. 10.3390/vaccines9010039 PMC7827578 33440895

[15] Mizumoto K Kagaya K Zarebski A Chowell G. Estimating the Asymptomatic Proportion of Coronavirus Disease 2019 (COVID-19) Cases on Board the Diamond Princess Cruise Ship, Yokohama, Japan, 2020. Eurosurveillance (2020) 25(10):2000180. 10.2807/1560-7917.ES.202025.10.2000180 PMC7078829 32183930

[16] Hu Z Song C Xu C Jin G Chen Y Xu X. Clinical Characteristics of 24 Asymptomatic Infections With COVID-19 Screened Among Close Contacts in Nanjing, China. Sci China Life Sci (2020) 63(5):706–11. 10.1007/s11427-020-1661-4 PMC7088568 32146694

[17] Simonnet A Chetboun M Poissy J Raverdy V Noulette J Duhamel A. High Prevalence of Obesity in Severe Acute Respiratory Syndrome Coronavirus-2 (SARS-CoV-2) Requiring Invasive Mechanical Ventilation. Obesity (2020) 28:1195–9. 10.1002/oby.22831 PMC7262326 32271993

[18] Assiri A Al-Tawfiq JA Al-Rabeeah AA Al-Rabiah FA Al-Hajjar S Al-Barrak A. Epidemiological, Demographic, and Clinical Characteristics of 47 Cases of Middle East Respiratory Syndrome Coronavirus Disease From Saudi Arabia: A Descriptive Study. Lancet Infect Dis (2013) 13(9):752–61. 10.1016/S1473-3099(13)70204-4 PMC7185445 23891402

[19] Honce R Schultz-Cherry S. Impact of Obesity on Influenza a Virus Pathogenesis, Immune Response, and Evolution. Front Immunol (2019) 10:1071(MAY). 10.3389/fimmu.2019.01071 31134099 PMC6523028

[20] Amirian ES. Potential Fecal Transmission of SARS-CoV-2: Current Evidence and Implications for Public Health. Int J Infect Dis (2020) 95:363–70. 10.1016/j.ijid.2020.04.057 PMC7195510 32335340

[21] Cheung KS Hung IF Chan PP Lung KC Tso E Liu R. Gastrointestinal Manifestations of SARS-CoV-2 Infection and Virus Load in Fecal Samples From the Hong Kong Cohort and Systematic Review and Meta-Analysis. Gastroenterology (2020). 10.1053/j.gastro.2020.03.065 PMC7194936 32251668

[22] Lamers MM Beumer J van der Vaart J Knoops K Puschhof J Breugem TI. SARS-CoV-2 Productively Infects Human Gut Enterocytes. Science (2020) 369:50–4. 10.1126/science.abc1669 PMC7199907 32358202

[23] Chen N Zhou M Dong X Qu J Gong F Han Y. Epidemiological and Clinical Characteristics of 99 Cases of 2019 Novel Coronavirus Pneumonia in Wuhan, China: A Descriptive Study. Lancet (2020) 395(10223):507–13. 10.1016/S0140-6736(20)30211-7 PMC7135076 32007143

[24] Ghosh S Klein RS. Sex Drives Dimorphic Immune Responses to Viral Infections. J Immunol (2017) 198(5):1782–90. 10.4049/jimmunol.1601166 PMC5325721 28223406

[25] Laffont S Seillet C Guéry JC. Estrogen Receptor-Dependent Regulation of Dendritic Cell Development and Function. Front Immunol (2017) 8:108(FEB). 10.3389/fimmu.2017.00108 28239379 PMC5300975

[26] Channappanavar R Fett C Mack M Ten Eyck PP Meyerholz DK Perlman S. Sex-Based Differences in Susceptibility to Severe Acute Respiratory Syndrome Coronavirus Infection. J Immunol (2017) 198(10):4046–53. 10.4049/jimmunol.1601896 PMC5450662 28373583

[27] Murphy AJ Guyre PM Pioli PA. Estradiol Suppresses NF-κB Activation Through Coordinated Regulation of Let-7a and miR-125b in Primary Human Macrophages. J Immunol (2010) 184(9):5029–37. 10.4049/jimmunol.0903463 PMC2882792 20351193

[28] Zhang X Wang L Zhang H Guo D Qiao Z Qiao J. Estrogen Inhibits Lipopolysaccharide-Induced Tumor Necrosis Factor-α Release From Murine Macrophages. Methods Find Exp Clin Pharmacol (2001) 23(4):169–73. 10.1358/mf.2001.23.4.634640 11676224

[29] Aomatsu M Kato T Kasahara E Kitagawa S. Gender Difference in Tumor Necrosis Factor-α Production in Human Neutrophils Stimulated by Lipopolysaccharide and Interferon-Γ. Biochem Biophys Res Commun (2013) 441(1):220–5. 10.1016/j.bbrc.2013.10.042 24140406

[30] Meier A Chang JJ Chan ES Pollard RB Sidhu HK Kulkarni S. Sex Differences in the Toll-Like Receptor-Mediated Response of Plasmacytoid Dendritic Cells to HIV-1. Nat Med (2009) 15(8):955–9. 10.1038/nm.2004 PMC2821111 19597505

[31] Cunningham MA. Estrogen Receptor Alpha Binding to ere is Required for Full Tlr7- and Tlr9-Induced Inflammation. SOJ Immunol (2014) 2(1):ew. 10.15226/soji.2014.00107 PMC4106444 25061615

[32] Qingxian C Fengjuan C Fang L Luo F Liu X Wu Q. Obesity and COVID-19 Severity in a Designated Hospital in Shenzhen, China. SSRN Electron J (2020). 10.2139/ssrn.3556658 32409502

[33] Nakamura K Fuster JJ Walsh K. Adipokines: A Link Between Obesity and Cardiovascular Disease. J Cardiol (2014) 63(4):250–9. 10.1016/j.jjcc.2013.11.006 PMC3989503 24355497

[34] van derVoort P Moser J Zandstra DF Kobold ACM Knoester M Calkhoven CF. Leptin Levels in SARS-Cov-2 Infection Related Respiratory Failure: A Cross-Sectional Study and a Pathophysiological Framework on the Role of Fat Tissue. Heliyon (2020) 6(8):e04696. 10.1016/j.heliyon.2020.e04696 32844126 PMC7439829

[35] Luzi L Radaelli MG. Influenza and Obesity: Its Odd Relationship and the Lessons for COVID-19 Pandemic. Acta Diabetol (2020) 57(6):759–64. 10.1007/s00592-020-01522-8 PMC7130453 32249357

[36] Leung C. Risk Factors for Predicting Mortality in Elderly Patients With COVID-19: A Review of Clinical Data in China. Mech Ageing Dev (2020) 188:111255. 10.1016/j.mad.2020.111255 32353398 PMC7184979

[37] Frasca D Blomberg BB. Inflammaging Decreases Adaptive and Innate Immune Responses in Mice and Humans. Biogerontology (2016) 17(1):7–19. 10.1007/s10522-015-9578-8 25921609 PMC4626429

[38] Milan-Mattos JC Anibal FF Perseguini NM Minatel V Rehder-Santos P Castro CA. Effects of Natural Aging and Gender on Pro-Inflammatory Markers. Braz J Med Biol Res (2019) 52(9):e8392–402. 10.1590/1414-431x20198392 PMC6694726 31411315

[39] Jiang F Deng L Zhang L Cai Y Cheung CW Xia Z. Review of the Clinical Characteristics of Coronavirus Disease 2019 (COVID-19). J Gen Intern Med (2020) 35(5):1545–9. 10.1007/s11606-020-05762-w PMC7088708 32133578

[40] Huang C Wang Y Li X Ren L Zhao J Hu Y. Clinical Features of Patients Infected With 2019 Novel Coronavirus in Wuhan, China. Lancet (2020) 395(10223):497–506. 10.1016/S0140-6736(20)30183-5 31986264 PMC7159299

[41] Giannis D Ziogas IA Gianni P. Coagulation Disorders in Coronavirus Infected Patients: COVID-19, SARS-CoV-1, MERS-CoV and Lessons From the Past. J Clin Virol (2020) 127:104362. 10.1016/j.jcv.2020.104362 32305883 PMC7195278

[42] Tang N Bai H Chen X Gong J Li D Sun Z. Anticoagulant Treatment is Associated With Decreased Mortality in Severe Coronavirus Disease 2019 Patients With Coagulopathy. J Thromb Haemost (2020) 18(5):1094–9. 10.1111/jth.14817 PMC9906401 32220112

[43] Aziz M Fatima R Assaly R. Elevated Interleukin-6 and Severe COVID-19: A Meta-Analysis. J Med Virol (2020) April):jmv.25948. 10.1002/jmv.25948 PMC7267383 32343429

[44] Land WG. Use of Damps and Samps as Therapeutic Targets or Therapeutics: A Note of Caution. Mol Diagn Ther (2020) 24:1–12. 10.1007/s40291-020-00460-z 32248387 PMC7127836

[45] Chen L DiPietro LA. Toll-Like Receptor Function in Acute Wounds. Adv Wound Care (2017) 6(10):344–55. 10.1089/wound.2017.0734 PMC5649397 29062591

[46] Zheng S Fan J Yu F Feng B Lou B Zou Q. Viral Load Dynamics and Disease Severity in Patients Infected With SARS-CoV-2 in Zhejiang Province, China, January-March 2020: Retrospective Cohort Study. BMJ (2020) 369:m1443. 10.1136/bmj.m1443 32317267 PMC7190077

[47] Van Der Made CI Simons A Schuurs-Hoeijmakers J Van Den Heuvel G Mantere T Kersten S. Presence of Genetic Variants Among Young Men With Severe COVID-19. JAMA - J Am Med Assoc (2020) 324(7):663–73. 10.1001/jama.2020.13719 PMC7382021 32706371

[48] Galluzzi L Kepp O Kroemer G. Mitochondria: Master Regulators of Danger Signalling. Nat Rev Mol Cell Biol (2012) 13(12):780–8. 10.1038/nrm3479 23175281

[49] Faust HE Reilly JP Anderson BJ Ittner CAG Forker CM Zhang P. Plasma Mitochondrial DNA Levels are Associated With ARDS in Trauma and Sepsis Patients. Chest (2020) 157(1):67–76. 10.1016/j.chest.2019.09.028 31622590 PMC6965693

[50] Figueroa-Lozano S Valk-Weeber RL Akkerman R Abdulahad W van Leeuwen SS Dijkhuizen L. Inhibitory Effects of Dietary N-Glycans From Bovine Lactoferrin on Toll-Like Receptor 8; Comparing Efficacy With Chloroquine. Front Immunol (2020) 11:790. 10.3389/fimmu.2020.00790 32477333 PMC7235371

[51] Lamphier M Zheng W Latz E Spyvee M Hansen H Rose J. Novel Small Molecule Inhibitors of TLR7 and TLR9: Mechanism of Action and Efficacy in Vivo. Mol Pharmacol (2014) 85(3):429–40. 10.1124/mol.113.089821 24342772

[52] Mallat J Hamed F Balkis M Mohamed MA Mooty M Malik A. Hydroxychloroquine Is Associated With Slower Viral Clearance in Clinical COVID-19 Patients With Mild to Moderate Disease. Med (Baltimore) (2020) 99(52):e23720. 10.1097/MD.0000000000023720 PMC7769326 33350752

[53] National Center for Biotechnology Information. Pubchem Compound Summary for CID 60961, Adenosine (2021). Available at: https://pubchem.ncbi.nlm.nih.gov/compound/Adenosine#section=Drug-and-Medication-Information (Accessed April 8, 2021).

[54] Silva L Subiabre M Araos J Sáez T Salsoso R Pardo F. Insulin/Adenosine Axis Linked Signalling. Mol Aspects Med (2017) 55:45–61. 10.1016/j.mam.2016.11.002 27871900

[55] Fredholm BB IJzerman AP Jacobson KA Linden J Müller CE. International Union of Basic and Clinical Pharmacologyand Classification of Adenosine Receptors—an Update. Pharmacol Rev (2011) 63(1):1–34. 10.1124/pr.110.003285 PMC3061413 21303899

[56] Borea PA Gessi S Merighi S Vincenzi F Varani K. Pharmacology of Adenosine Receptors: The State of the Art. Physiol Rev (2018) 98:1591–625. 10.1152/physrev.00049.2017.-Adenosine 29848236

[57] Young JD Yao SYM Baldwin JM Cass CE Baldwin SA. The Human Concentrative and Equilibrative Nucleoside Transporter Families, SLC28 and SLC29. Mol Aspects Med (2013) 34(2-3):529–47. 10.1016/j.mam.2012.05.007 23506887

[58] FDA. ADENOSCAN ® (Adenosine Injection) for INTRAVENOUS INFUSION ONLY Description Clinical Pharmacology Mechanism of Action. Available at: https://www.accessdata.fda.gov/drugsatfda_docs/label/2009/020059s014lbl.pdf (Accessed June 10, 2020).

[59] FDA. Lexiscan (Regadenoson) Label. Available at: www.fda.gov/medwatch (Accessed June 10, 2020).

[60] Majumdar S Aggarwal BB. Adenosine Suppresses Activation of Nuclear Factor-κb Selectively Induced by Tumor Necrosis Factor in Different Cell Types. Oncogene (2003) 22(8):1206–18. 10.1038/sj.onc.1206184 12606947

[61] Varani K Padovan M Vincenzi F Targa M Trotta F Govoni M. A2A and A3 Adenosine Receptor Expression in Rheumatoid Arthritis: Upregulation, Inverse Correlation With Disease Activity Score and Suppression of Inflammatory Cytokine and Metalloproteinase Release. Arthritis Res Ther (2011) 13(6):R197–210. 10.1186/ar3527 PMC3334647 22146575

[62] Pinhal-Enfield G Ramanathan M Hasko G Vogel SN Salzman AL Boons G-J. An Angiogenic Switch in Macrophages Involving Synergy Between Toll-Like Receptors 2, 4, 7, and 9 and Adenosine A2A Receptors. Am J Pathol (2003) 163(2):711–21. 10.1016/S0002-9440(10)63698-X PMC1868201 12875990

[63] Campo GM Avenoso A D’Ascola A Scuruchi M Prestipino V Nastasi G. Adenosine A2A Receptor Activation and Hyaluronan Fragment Inhibition Reduce Inflammation in Mouse Articular Chondrocytes Stimulated With Interleukin-1β. FEBS J (2012) 279(12):2120–33. 10.1111/j.1742-4658.2012.08598.x 22502642

[64] Ferrari D Wesselborg S Bauer MKA Schulze-Osthoff K. Extracellular ATP Activates Transcription Factor NF-κB Through the P2Z Purinoreceptor by Selectively Targeting NF-κB P65 (Rela). J Cell Biol (1997) 139(7):1635–43. 10.1083/jcb.139.7.1635 PMC2132650 9412459

[65] He Y Franchi L Núñez G. TLR Agonists Stimulate Nlrp3-Dependent IL-1β Production Independently of the Purinergic P2X7 Receptor in Dendritic Cells and in Vivo. J Immunol (2013) 190(1):334–9. 10.4049/jimmunol.1202737 PMC3531812 23225887

[66] Zhao R Qiao J Zhang X Zhao Y Meng X Sun D. Toll-Like Receptor-Mediated Activation of CD39 Internalization in Bmdcs Leads to Extracellular ATP Accumulation and Facilitates P2X7 Receptor Activation. Front Immunol (2019) 10:2524(OCT). 10.3389/fimmu.2019.02524 31736956 PMC6834529

[67] Haskó G. Adenosine: An Endogenous Regulator of Innate Immunity. Trends Immunol (2004) 25(1):33–9. 10.1016/j.it.2003.11.003 14698282

[68] Sun Y Duan Y Eisenstein AS Hu W Quintana A Lam WK. A Novel Mechanism of Control of Nfκb Activation and Inflammation Involving A2B Adenosine Receptors. J Cell Sci (2012) 125(19):4507–17. 10.1242/jcs.105023 PMC3500865 22767505

[69] Haskó G Pacher P. A 2A Receptors in Inflammation and Injury: Lessons Learned From Transgenic Animals. J Leukoc Biol (2008) 83(3):447–55. 10.1189/jlb.0607359 PMC2268631 18160539

[70] Lee HT Kim M Joo JD Gallos G Chen J-F Emala CW. A 3 Adenosine Receptor Activation Decreases Mortality and Renal and Hepatic Injury in Murine Septic Peritonitis. Am J Physiol Integr Comp Physiol (2006) 291(4):R959–69. 10.1152/ajpregu.00034.2006 16728466

[71] Gallos G Ruyle TD Emala CW Lee HT. A 1 Adenosine Receptor Knockout Mice Exhibit Increased Mortality, Renal Dysfunction, and Hepatic Injury in Murine Septic Peritonitis. Am J Physiol Physiol (2005) 289(2):F369–76. 10.1152/ajprenal.00470.2004 15784841

[72] Moore CC Martin EN Lee GH Obrig T Linden J Scheld WM. An A2Aadenosine Receptor Agonist, ATL313, Reduces Inflammation and Improves Survival in Murine Sepsis Models. BMC Infect Dis (2008) 8(1):141–51. 10.1186/1471-2334-8-141 PMC2588444 18937852

[73] Zhong H Belardinelli L Maa T Feoktistov I Biaggioni I Zeng D. A2B Adenosine Receptors Increase Cytokine Release by Bronchial Smooth Muscle Cells. Am J Respir Cell Mol Biol (2004) 30(1):118–25. 10.1165/rcmb.2003-0118OC 12855406

[74] Varani K Caramori G Vincenzi F Adcock I Casolari P Leung E. Alteration of Adenosine Receptors in Patients With Chronic Obstructive Pulmonary Disease. Am J Respir Crit Care Med (2006) 173(4):398–406. 10.1164/rccm.200506-869OC 16322645

[75] Brown RA Clarke GW Ledbetter CL Hurle MJ Denyer JC Simcock DE. Elevated Expression of Adenosine A1 Receptor in Bronchial Biopsy Specimens From Asthmatic Subjects. Eur Respir J (2008) 31(2):311–9. 10.1183/09031936.00003707 17959644

[76] Le T-TT Berg NK Harting MT Li X Eltzschig HK Yuan X. Purinergic Signaling in Pulmonary Inflammation. Front Immunol (2019) 10:1633. 10.3389/fimmu.2019.01633 31379836 PMC6646739

[77] Schepp CP Reutershan J. Bench-to-Bedside Review: Adenosine Receptors – Promising Targets in Acute Lung Injury? Crit Care (2008) 12(5):226. 10.1186/cc6990 18828873 PMC2592730

[78] Reutershan J Cagnina RE Chang D Linden J Ley K. Therapeutic Anti-Inflammatory Effects of Myeloid Cell Adenosine Receptor A2a Stimulation in Lipopolysaccharide-Induced Lung Injury. J Immunol (2007) 179(2):1254–63. 10.4049/jimmunol.179.2.1254 17617618

[79] Kutzsche S Lyberg T Bjertnaes LJ. Effects of Adenosine on Extravascular Lung Water Content in Endotoxemic Pigs. Crit Care Med (2001) 29(12):2371–6. 10.1097/00003246-200112000-00021 11801842

[80] Eckle T Hughes K Ehrentraut H Brodsky KS Rosenberger P Choi D. Crosstalk Between the Equilibrative Nucleoside Transporter ENT2 and Alveolar Adora2b Adenosine Receptors Dampens Acute Lung Injury. FASEB J (2013) 27(8):3078–89. 10.1096/fj.13-228551 PMC3714574 23603835

[81] Chambers ED White A Vang A Wang Z Ayala A Weng T. Blockade of Equilibrative Nucleoside Transporter 1/2 Protects Against Pseudomonas Aeruginosa– Induced Acute Lung Injury and NLRP3 Inflammasome Activation. FASEB J (2020) 34(1):1516–31. 10.1096/fj.201902286R PMC7045807 31914698

[82] Gonzales JN Gorshkov B Varn MN. Protective Effect of Adenosine Receptors Against Lipopolysaccharide-Induced Acute Lung Injury. Am J Physiol Cell Mol Physiol (2014) 306(6):L497–507. 10.1152/ajplung.00086.2013 PMC3949083 24414256

[83] Sharma AK Linden J Kron IL Laubach VE. Protection From Pulmonary Ischemia-Reperfusion Injury by Adenosine A2A Receptor Activation. Respir Res (2009) 10(1):58–67. 10.1186/1465-9921-10-58 19558673 PMC2711962

[84] Ellman PI Reece TB Law MG Linden J Tribble CG Kron IL. Adenosine A2a Activation Attenuates Nontransplantation Lung Reperfusion Injury. J Surg Res (2008) 149(1):3–8. 10.1016/j.jss.2007.08.008 17937935

[85] Lisle TC Gazoni LM Fernandez LG Sharma AK Bellizzi AM Schifflett GD. Inflammatory Lung Injury After Cardiopulmonary Bypass is Attenuated by Adenosine A2A Receptor Activation. J Thorac Cardiovasc Surg (2008) 136(5):1280–8. 10.1016/j.jtcvs.2008.07.010 PMC2652167 19026816

[86] Sakamaki F Ishizaka A Urano T Sayama K Nakamura H Tarashima T. Attenuation by Intravenous 2-Chloroadenosine of Acute Lung Injury Induced by Live Escherichia Coli or Latex Particles Added to Endotoxin in the Neutropenic State. J Lab Clin Med (2003) 142(2):128–35. 10.1016/S0022-2143(03)00105-7 12960960

[87] Reece TB Ellman PI Maxey TS Crosby IK Warren PS Chong TW. Adenosine A2A Receptor Activation Reduces Inflammation and Preserves Pulmonary Function in an *in Vivo* Model of Lung Transplantation. J Thorac Cardiovasc Surg (2005) 129(5):1137–43. 10.1016/j.jtcvs.2004.11.042 15867791

[88] Correale P Caracciolo M Bilotta F Conte M Cuzzola M Falcone C. Therapeutic Effects of Adenosine in High Flow 21% Oxygen Aereosol in Patients With Covid19-Pneumonia. PloS One (2020) 15:e0239692. 10.1371/journal.pone.0239692 33031409 PMC7544127

[89] Caracciolo M Correale P Mangano C Foti G Falcone C Macheda S. Efficacy and Effect of Inhaled Adenosine Treatment in Hospitalized COVID-19 Patients. Front Immunol (2021) 12:613070. 10.3389/fimmu.2021.613070 33815368 PMC8012541

[90] Geiger JD Khan N Murugan M Boison D. Possible Role of Adenosine in COVID-19 Pathogenesis and Therapeutic Opportunities. Front Pharmacol (2020) 11:594487. 10.3389/FPHAR.2020.594487 33324223 PMC7726428

